# Intramammary Immunisation Provides Short Term Protection Against *Mannheimia haemolytica* Mastitis in Sheep

**DOI:** 10.3389/fvets.2021.659803

**Published:** 2021-06-10

**Authors:** Riccardo Tassi, Martina Schiavo, Joel Filipe, Helen Todd, David Ewing, Keith T. Ballingall

**Affiliations:** ^1^Department of Disease Control, Moredun Research Institute, Penicuik, United Kingdom; ^2^Biomathematics and Statistics Scotland, Edinburgh, United Kingdom

**Keywords:** mastitis, ovine, *Mannheimia haemolytica*, intramammary immunisation, Interleukin-17A

## Abstract

Mastitis affects both dairy and meat/wool sheep industries with losses due to reductions in milk quality and quantity, increased treatment costs and restricted lamb growth. Effective vaccines would be important tools for mastitis control. However, the development of vaccines against mastitis has proved challenging due to the failure to target protective immunity to the mammary gland. In order to target responses to the mammary gland, this study tested whether local administration directly into the gland through the teat canal or in the udder skin confers protection against an intramammary infection. In this study, we tested a vaccine that confers protection against respiratory disease caused by *Mannheimia haemolytica* to determine if it also protects against intramammary infection by the same organism. No evidence of protection was observed in animals that received a subcutaneous immunisation in the udder skin, however, intramammary immunisation provided almost complete protection against an experimental challenge administered 7 days post immunisation but not if the challenge was delivered 14 days post immunisation. To investigate further the nature of this variation in response, the somatic cell count and concentration of cytokines Interleukin-1β, Interleukin-10 and Interleukin-17A was determined in milk over the course of each study. Intramammary immunisation induced an inflammatory response within the mammary gland, characterised by increases in SCC and in the production of cytokines IL-1β, IL-10, and IL-17A. This response was similar to that observed in un-vaccinated control animals post challenge. The SCC and cytokine levels had returned to levels comparable with un-vaccinated controls prior to challenge at both 7 and 14 days post immunisation. The transient nature of the protective effect is consistent with the priming of an innate antibacterial response within the mammary gland which provides protection against challenge at 7 days but is diminished by 14 days post-vaccination. Further studies are planned to determine the nature of the innate immune mechanisms associated with the protective effect described here to determine whether it may be exploited to improve ruminant udder health.

## Introduction

Mastitis or inflammation of the mammary gland is most often caused by an intramammary infection (IMI) by a range of Gram-positive and negative species of bacteria. IMIs impact both dairy and meat/fibre sheep production systems worldwide through reduction in the quantity and quality of milk production. In suckling flocks the consequence of an IMI is a slowing of the growth rate of lambs with a corresponding increase in the time required to achieve target weights (1). Mastitis appears in a number of forms including a long lasting subclinical inflammatory form or a more acute clinical form which is a painful inflammatory condition with clear animal welfare implications and which may lead to the death or premature culling of animals from affected flocks (2). Accurate estimates of the economic losses attributed to ovine mastitis are not available, although it has been calculated in the UK alone that a reduction of just 10% in ovine mastitis cases would save £2.7 million per annum (3).

The most common bacterial species associated with ovine mastitis are *Staphylococcus aureus, Streptococcus uberis, Mannheimia haemolytica*, and several species of coagulase negative staphylococci (CNS) (4, 5). In meat production systems *M. haemolytica* is one of the most frequently identified causes of mastitis (5–8).

Several risk factors that predispose animals to mastitis have been identified. These include poor conformation of the udder, teat lesions, litter size (two or more lambs), poor body condition of the ewe and previous cases of mastitis (9). Suckling lambs have also been identified as a possible source of intramammary infections as young animals can carry the *M. haemolytica* bacteria in the upper respiratory tract (10).

As with cattle dairy, control of mastitis in sheep dairy systems relies on reducing the impact of bacterial contamination of the milking environment and rapid treatment of clinical cases. If implemented effectively such measures can substantially reduce the impact of mastitis in a flock (11). However, in more extensive sheep meat and fibre production systems the options available to the farmer are more limited. These include culling of old or previously affected ewes and breeding for an udder conformation that minimises the risk of teat damage and contamination of the teat apex (9, 12). The treatment of infected animals relies on antimicrobial drugs. Although the usage of antimicrobials in sheep farming does not appear to be as high as in other farmed animals (13) it may still represent a risk for the induction of antimicrobial resistance. Thus, there is a requirement to reduce the use of antimicrobials and the development of alternative mastitis prevention strategies including vaccines (14).

Despite many attempts at developing mastitis vaccines for dairy ruminant species, few are currently available and all target disease in dairy cattle. Notable examples include vaccines against *Escherichia coli* (15) and *S. aureus* (16). As these vaccines appear to reduce the clinical symptoms of mastitis but not the infection rate, current research is focused on vaccines that induce not only a strong humoral response but also a cellular response within the mammary gland (17). Recent studies suggest that a cellular response may be key in clearing an intramammary infection (17–19), specifically a Th17 type response (20, 21). The Th17 response targets extracellular bacteria by enhancing innate immune mechanisms such as phagocyte activity and the production of antimicrobial peptides by mammary epithelial cells (22). Also several studies suggest that delivery of the vaccine directly into the mammary gland as opposed to a systemic route, may enhance its efficacy (23–25).

Vaccines which protect sheep from the respiratory disease caused by *M. haemolytica* have been available for many years. Such vaccines consist of several serotypes of *M. haemolytica* grown in iron deficient medium. This induces expression of iron regulated proteins on the surface of the bacteria (26). These proteins are immunogenic and induce a protective response in immunised animals. The vaccine is generally administered to pregnant ewes in order to induce colostral antibodies which protect their lambs during the first few weeks of their life. Despite its routine use there are no studies reported which investigate the potential of these vaccines to protect against mastitis caused by *M. haemolytica*. Therefore, the aim of this study was to test whether a vaccine originally developed against the respiratory disease caused by *M. haemolytica* also provides protection against mastitis when administered in the mammary gland's subcutaneous tissue or directly infused into the mammary gland through the teat canal.

## Materials and Methods

### Animals

Lactating Scottish Mules between 2 and 6 years old and ~1 month into lactation were sourced from the Moredun Research Institute (MRI) flock. Prior to enrolling into each study, milk samples from each mammary half were screened for pre-existing intramammary infection by bacterial culture as described by Zadoks et al. (5). Animals were housed with their lambs in loose pens with straw bedding and had access to water and grass hay *ad libitum* and concentrate was fed twice daily. After weaning animals were hand milked once (study 1) or twice (studies 2 and 3) each day to maintain lactation. All studies were conducted under UK Government Home Office licence following approval of the MRI Animal Welfare and Ethical Review Body (AWERB) in accordance with the Animals (Scientific Procedures) Act 1986.

### Immunisation Regime

**Figure 1:**
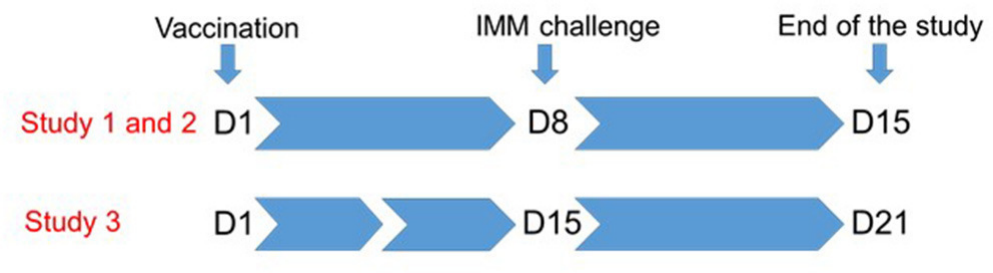
Overview of the study design. Lambs were weaned from their mothers 24 h prior to immunisation at D1. Each ewe was immunised subcutaneously in the udder skin (study 1) or directly into the mammary gland through the teat canal (studies 1, 2, and 3). Animals were challenged by infusion of the teat canal with *M. haemolytica* at D8 (study 1 and 2), or at D15 days (study 3). Animals were followed for 7 days after challenge.

We conducted three studies to test whether local administration of the Ovipast plus vaccine (MSD animal health, Milton Keynes, UK) confers protection against an intramammary infection caused by *M. haemolytica*. The Ovipast plus vaccine includes several serotypes of *M. haemolytica* and *Bibersteinia threalhosi* grown in an iron deficient medium prior to formalin inactivation. Aluminium hydroxide is used as an adjuvant with this vaccine preparation. As part of standard flock management, this vaccine is used in the control of the systemic and respiratory disease caused by these bacterial species in ewes and lambs. All ewes are immunised between 4 and 6 weeks prior to parturition by subcutaneous injection in the lateral side of the upper neck, so are not immunologically naïve. In our studies, animals were boosted with the same vaccine 24 h after weaning by one of the two routes detailed below. An overview of the time line of each study is described in Figure 1.

#### Study 1

The effect of local immunisation with the Ovipast vaccine when delivered directly into the mammary gland through the teat canal or subcutaneously over the supramammary lymph node was tested in study 1 in 29 sheep. Both mammary halves were stripped of milk and the teat ends disinfected with cotton wool swabs soaked in 70% (vol/vol) ethanol. Two milliliters of vaccine were administrated subcutaneously each side of the mammary gland, ~5 cm from the supramammary lymph node (*n* = 10) or directly into the mammary gland (*n* = 10) using a J-12 teat infusion cannula (Jorgens Laboratories, Loveland, USA). The control group (*n* = 9), did not receive the vaccine. One animal was removed from the control group due to an unrelated IMI detected prior to immunisation. The efficacy of the vaccine was tested after 7 days by infusion of the teat canal with *M. haemolytica*.

#### Study 2

Study 2 was conducted to ensure reproducibility of the intramammary vaccination data observed in the first study. In study 2, group sizes were reduced to five animals based on power calculations using data from study 1 in accordance with the principles of the 3Rs (27). Two milliliters of vaccine were administrated directly into the mammary gland via a teat infusion cannula (*n* = 5). The control group (*n* = 5) did not receive the vaccine.

#### Study 3

Study 3 was conducted to test the efficacy of intramammary vaccination 14 days after administration. In this study, a total of 10 animals were used in two groups of 5 animals. Two milliliters of vaccine were administrated directly into the mammary gland via a teat infusion cannula in the experimental group (*n* = 5) and the control group (*n* = 5) did not receive the vaccine.

### Intramammary Challenge With *Mannheimia haemolytica*

To test whether immunisation with the Ovipast vaccine confers protection against mastitis, all animals were challenged directly into the mammary gland via the teat canal with *M. haemolytica* FSL T1-008. FSL T1-008 was isolated from a case of sheep mastitis (5) and in our preliminary study it was shown to cause clinical mastitis in lactating ewes when infused into the mammary gland via the teat canal (data not shown). Animals were challenged in both mammary halves with ~2,000 cfu. The challenge was delivered either 7 days (studies 1 and 2) or 14 days (study 3) after immunisation (Figure 1).

#### Preparation and Administration of the Bacterial Challenge

The bacterial inoculum was prepared from stock cultures stored at −80°C. A stock culture was thawed, plated on 5% sheep blood agar (E&O Laboratories, Bonnybridge, UK) and incubated overnight at 37°C to check for viability and colony purity. Two to five colonies were inoculated into 45 ml of nutrient broth (Difco, Cambridge, UK) and incubated for 14 h at 37°C with 150 rpm shaking. Based on preliminary experiments the bacterial suspension was diluted in sterile phosphate buffered saline (PBS) to the target concentration of ~1,000 cfu/ml. The actual inoculum concentration was checked by the viable count method. A series of 10-fold dilutions were prepared in sterile PBS and three 10 μl drops per dilution plated onto horse blood agar plates (E&O Laboratories) and incubated overnight at 37°C. Colonies were counted when they were in the range of 5–50 cfu per spot, and the bacterial concentration for each time point (cfu/ml) was calculated based on average colony counts for the appropriate dilution. Prior to challenge, sheep were milked, the teat ends disinfected and 2 ml of inoculum (target dose: 2,000 cfu/mammary half) infused into each mammary half. After infusion sheep were returned to their pen.

### Data and Sample Collection

Milk samples collected throughout the study were used for qualitative and quantitative bacteriology, measurement of the somatic cell count (SCC) and quantification of pro and anti-inflammatory cytokines levels. Milk samples for bacteriological analysis were collected using aseptic technique (28) and stored on ice until refrigeration at 4°C.

### Qualitative and Quantitative Bacteriology Analysis

For qualitative bacteriology, 10 μl of milk from each mammary half were plated on 5% horse or sheep blood agar (E&O Laboratories) and incubated at 37°C for 24 h. Identification of bacterial species was based on morphology and standard biochemical tests including Gram staining, catalase test, and esculin splitting. For quantitative analysis, milk samples were serially diluted 10-fold in sterile PBS. Triplicate 10 μl aliquots of each dilution were spotted on blood agar plates (E&O Laboratories), allowed to air dry and incubated overnight at 37°C. Colonies with morphology consistent with *M. haemolytica* were counted, if possible for the dilution showing between 5 and 50 cfu per spot. The bacterial concentration in milk (cfu/ml) was calculated based on average colony counts for appropriate dilutions.

### SCC and Preparation of Skimmed Milk for Cytokine Analysis

Approximately 35 ml of milk from each mammary half was collected in a 50 ml Falcon tube (Corning, Amsterdam, The Netherlands) and stored at 4°C until analysis. Five millilitres of milk was used for SCC no later than 48 h after collection using a DeLaval DCC Cell counter (DeLaval, Cardiff, UK). Samples were diluted up to 10-fold in PBS when the cellular concentration in the neat sample was beyond the detection limit. Cell count data is presented as cells/ml. The remaining 30 ml of milk were centrifuged at 3,500 × g at 4°C for 20 min. The fat layer was discarded and the supernatant was transferred to a new 50 ml Falcon tube. Centrifugation was repeated and the supernatant stored at −80°C.

### Measurement of Cytokines in Milk

The concentration of cytokines Interleukin-1β (IL-1β), Interleukin-10 (IL-10) and Interleukin-17A (IL-17A) were measured in skimmed milk samples by sandwich ELISA tests using the antibodies detailed in Table 1. Microtitre plates (Immunolon 2 HB, Thermo Electron Corporation, Langenselbold, Germany for IL-1β and IL-10 ELISAs and Elisa Medium Binding M129A, Greiner Bio-One, Kremsmünster, Austria for the IL-17A ELISA) were coated overnight at 4°C with 100 μl/well of the appropriate coating antibody in 0.5 M carbonate buffer (0.5 M Na_2_CO_3_, 0.5 M NaHCO_3_, pH 9.6) at concentrations detailed in Table 1. Wells were washed with washing buffer (PBS, pH 7.4 and 0.05% vol/vol Tween 20) and non-specific binding sites blocked with 300 μl/well of PBS containing 3% (wt/vol) BSA and 0.05% (vol/vol) Tween 20 at room temperature for 1 h. Plates were washed and incubated for 1 h at room temperature with 100 μl/well of skimmed milk. Each sample was tested in duplicate. When necessary, samples were diluted with PBS supplemented with 0.05% (vol/vol) Tween 20 and 1% (wt/vol) BSA (reagent diluent). A standard curve of known cytokine concentrations was determined using dilutions of appropriate standards. Recombinant bovine standards were used for IL-1β (Bio-Rad-antibodies), and IL-17A (Kinghfisher Biotech, Saint Paul, USA). For IL-10 a recombinant ovine standard was provided by Sean Wattegedera (MRI, UK). ELISA plates were washed and 100 μl/well of detection antibody (Table 1) added, followed by incubation for 1 h at room temperature. For IL-10 and IL-17A, 100 μl/well of horseradish peroxidase (HRP)-streptavidin (Sigma-Aldrich) diluted 1:500 in reagent diluent were added, followed by incubation for 45 min at room temperature. IL-1β plates were incubated with 100 μl/well of HRP-conjugated polyclonal goat anti-rabbit immunoglobulins (Dako, Ely, UK) diluted 1:1,000 in reagent diluent for 1 h at room temperature. After incubation with HRP-streptavidin or HRP-conjugated antibody, plates were washed and incubated for 20 min at room temperature with 100 μl per well of o-Phenylenediamine dihydrochloride substrate (Sigma-Aldrich). The reaction was stopped with 25 μl per well of 2.5 M H_2_SO_4_ and optical density measured at 492 nm using a sunrise absorbance reader (Tecan, Theale, UK). Cytokine concentrations in skimmed milk samples were then calculated from the standard curve.

**Table 1:** Details of antibodies used for cytokine ELISAs.

**Target**	**Antibody**	**Isotype, Origin**	**Conc. (μg/ml)**	**Source**
Ovine IL-1β Coating	1D4	IgG1, mouse	1	Bio-Rad-Antibodies
Ovine IL-1β Detection	Polyclonal	n/a, rabbit	2	Bio-Rad-Antibodies
Bovine IL-10 Coating	CC318	IgG2b, mouse	4	Bio-Rad-Antibodies
Bovine IL-10 Detection	Biotinylated CC320	IgG1, mouse	1	Bio-Rad-Antibodies
Bovine IL-17A Coating	Polyclonal	n/a, rabbit	2	Kingfisher Biotech
Bovine IL-17A Detection	Biotinylated polyclonal	n/a, rabbit	1	Kingfisher Biotech

### Statistical Analysis

The effect of vaccination on bacterial counts, SCC and cytokine concentration was assessed using mixed models, which account for correlation between repeated observations from the same animal. The data from studies 1 and 2 were analysed together, as inoculation and challenge were administered at the same time in each experiment, with a random intercept included to account for potential differences between the two studies. Data from study 3 was analysed separately, as the time of challenge was different in this study. In all models, time, treatment and the interaction between time and treatment were considered as potential explanatory variables (in both zero and non-zero parts of zero-inflated models) and random intercepts were included for each animal. Model selection was carried out using likelihood ratio tests (LRTs) with final models fitted using restricted maximum likelihood (REML). The false discovery rate method was used to adjust for multiple comparisons across levels of several factors. All statistical analyses were conducted in R 4.0.2 (29). The models fitted to each response variable are explained in more detail below.

Bacterial counts were transformed (ln+1) and analysed using a zero-inflated linear mixed model (LMM) with random intercepts considered to account for differences between experiments 1 and 2 and differences between animals. A zero-inflated LMM was used to account for the fact that a large proportion (63%) of the non-missing values following challenge were zero. By fitting a zero-inflated model we fit models to the zero-generating process and the non-zero values separately. The models were fitted using the R package glmmTMB (30).

SCC data were transformed (ln) and analysed using generalised additive mixed models (GAMMs) fitted to the log of SCC. GAMMs fit smooth splines to capture potentially nonlinear relationships between the explanatory variables (time and treatment) and the response (SCC). GAMMs were fitted using the mgcv package in R (31). The fitted model included separate thin plate splines fitted to each treatment group and separate factor smooth splines with equivalent levels of smoothness fitted to each animal to account for variability in the trajectories of individual animals.

A linear mixed effects model was also fitted to the log-transformed SCC data to check for statistically significant differences specifically at time point 8 (immediately pre-challenge) in studies 1 and 2 and at time point 15 in study 3.

Cytokine concentrations were transformed (ln+1) and analysed using (zero-inflated linear) mixed models, as for bacterial counts. Given the clear non-linearity in data a GAMM was considered, however there were insufficient time points to fit such a model. Consequently, time was included as a categorical (factor) variable in the analysis and comparisons between treatment groups were made at each time point. To minimise the loss in power associated with multiple comparisons and to avoid issues arising from complete separation (all responses as zero or non-zero in some groups), the measurements taken before challenge were assigned to a “pre-challenge” category (rather than carrying out separate comparisons at the four pre-challenge timepoints). Consequently, there were five timepoints considered in each study (pre-challenge, day 9, 10, 12, and 14 for studies 1 and 2 and pre-challenge, day 16, 17, 19, and 21 for study 3).

For IL-1β and IL-17A a zero-inflated LMM was used, as for bacterial counts. For IL-10 data from studies 1 and 2 a zero-inflated generalised LMM with a Tweedie error distribution was used. The Tweedie distribution was chosen to better satisfy the assumptions regarding the distribution of residuals, as the non-zero values were typically small. A standard LMM resulted in confidence intervals for estimated IL-10 concentrations that included negative values (which was nor observed under a Tweedie distribution). In the case of the IL-10 data from experiment 3 a LMM with no zero-inflation component was fitted to only the data from day 16 onwards as the data prior to this was almost exclusively zero (creating model fitting issues due to complete separation) but from day 16 onwards there were few zeroes.

The output of the models used is shown in Supplementary Tables 1–10.

## Results

### Clinical and Bacteriology Data

**Figure 2:**
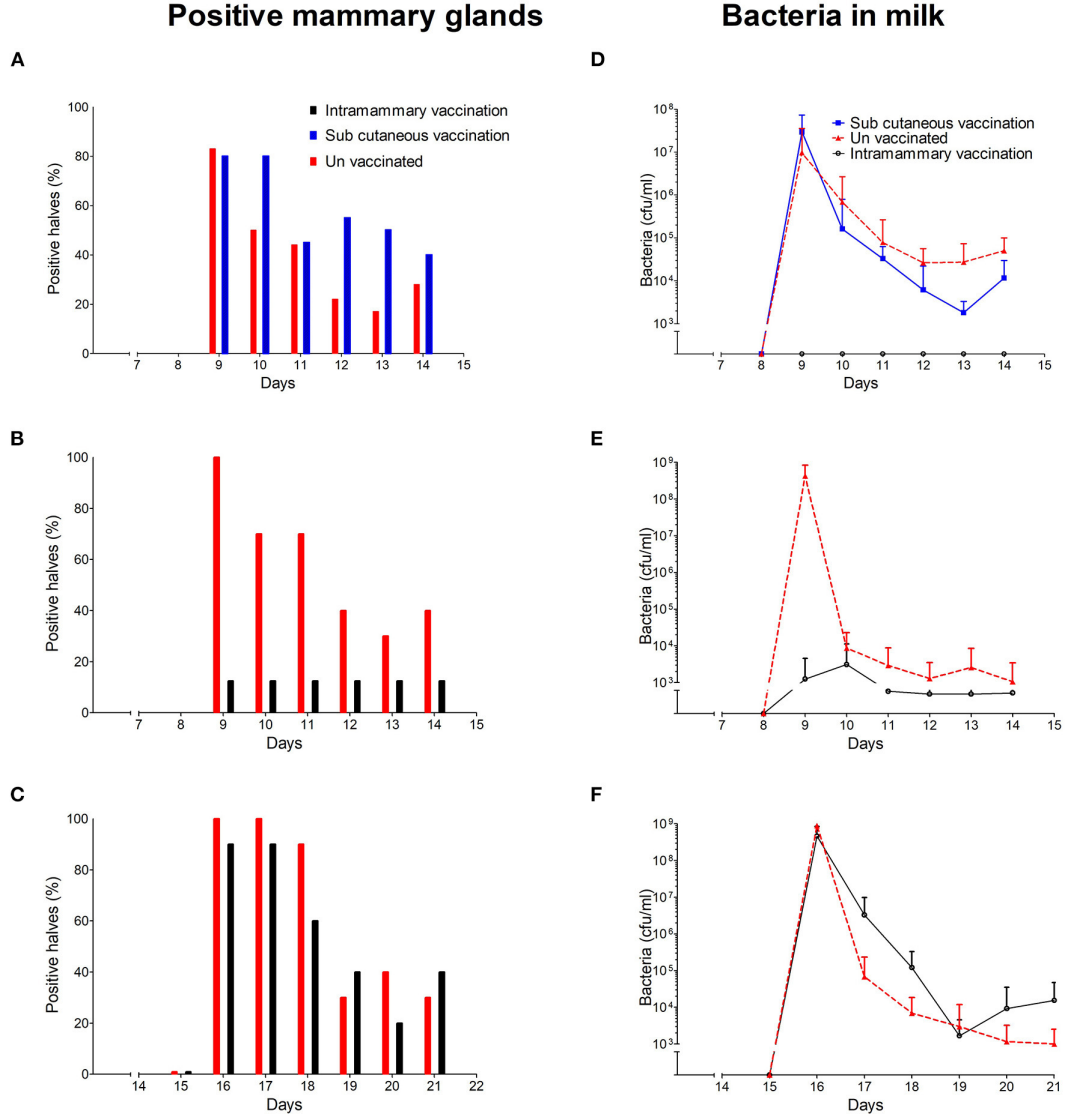
The percentage of *M. haemolytica* positive mammary glands in study 1 **(A)**, study 2 **(B)**, and study 3 **(C)**. The concentration of *M. haemolytica* in milk from study 1 **(D)**, study 2 **(E)**, and study 3 **(F)**. Average bacterial concentrations with standard deviations are shown.

To test whether immunisation targeted to the mammary gland provided protection against mastitis caused by *M. haemolytica*, we immunised and challenged groups of sheep in a series of studies over a 2 year period. All unvaccinated control animals developed an IMI in one or both mammary halves following intramammary *M. haemolytica* challenge. In total, 15 of the 18 control mammary halves were bacteriologically positive 24 h post challenge (PC) in study 1 (Figure 2A) and all 10 control mammary halves were bacteriologically positive 24 h PC in studies 2 and 3 (Figures 2B,C). The number of the infected mammary halves in control animals showed a reduction from 48 h PC. By the end of the study, 5/18, 4/10, and 3/10 halves remained bacteria positive in the control groups of all three studies, respectively (Figures 2A–C).

In the first study we tested two vaccine delivery routes, subcutaneous in the udder skin and an intramammary administration directly into the mammary gland via the teat canal. No evidence of early protection against challenge was observed in animals that received the subcutaneous vaccination, with 16 of the 20 mammary halves infected at 24 h PC (Figure 2A). In contrast to the subcutaneous and control groups, all intramammary immunised animals appeared fully protected at 24 h PC. This group remained protected until the end of the study 1 week PC (Figure 2A). On repeating this in study 2, an additional five animals received the vaccine via the intramammary route. Of these, one of the 10 challenged halves developed an infection (Figure 2B). In total, of the 30 mammary halves in studies 1 and 2 that received the vaccine via the intramammary route only 1 developed an infection at 24 h PC compared to 25 of the 28 control halves. However, in the third study when the challenge was delivered 2 weeks post immunisation this impressive level of protection was not observed. On this occasion, 9 of the 10 mammary halves became infected (Figure 2C).

The quantitative analysis of bacteria in milk samples from control animals peaked at 24 h in all three studies (Figures 2D–F). Bacterial counts gradually decreased thereafter with several control animals clearing the infection by the end of the study (Figures 2D–F).

Of the animals immunised directly into the mammary gland through the teat canal in study 1, very few bacteria were recovered in milk samples after challenge (Figure 2D). The mean bacterial concentration across the intramammary immunised animals in study 2 reached a peak of 3.13 × 10^3^ ± 8.27 × 10^3^ cfu/ml 48 h PC (Figure 2E) 13.8 × 10^3^ fold lower than the control values of 4.34 × 10^8^ ± 4.04 × 10^8^.

The bacterial concentration in milk samples from animals subcutaneously immunised in study 1 followed a similar pattern to those observed in un-vaccinated control animals. However, the peak bacterial concentration (2.95 × 10^7^ ± 4.37 × 10^7^ cfu/ml) at 24 h PC was ~14 fold lower than the peak concentration of 4.34 × 10^8^ ± 4.04 × 10^8^ observed in the unvaccinated control group at the same time point (Figure 2D).

The final fitted LMM for studies 1 and 2 included time after challenge and a quadratic term for time after challenge as fixed effects in the non-zero model and treatment and time after challenge as fixed effects in the zero model. Random intercepts for animal were included in both parts of the model and a random intercept for experiment was included in the non-zero part of the model. Both the zero and non-zero parts of the model indicate that subcutaneous vaccination did not significantly reduce the *S. uberis* bacterial concentration compared to the non-immunised controls (Supplementary Table 1). However, there was strong evidence that the intramammary vaccine reduced the number of animals shedding bacteria after challenge (*p* < 0.001).

In contrast to animals in studies 1 and 2 which were immunised and challenged after 1 week, bacterial concentrations in milk samples from animals challenged 2 weeks post vaccination followed a similar pattern to that observed in the control animals (Figure 2F). In this group the maximal mean bacterial concentration of 9 × 10^8^ ± 2 × 10^8^ cfu/ml was observed 24 h PC, a concentration ~2-fold higher than that observed in control animals (4.1 × 10^8^ ± 1.5 × 10^8^). Thereafter, the average bacterial concentration in milk decreased. The statistical analysis showed no differences in the mean concentration between the vaccinated and the control group both in the count and zero inflated part of the model (supplementary Table 2).

### Somatic Cell Response to Immunisation and Challenge

**Figure 3:**
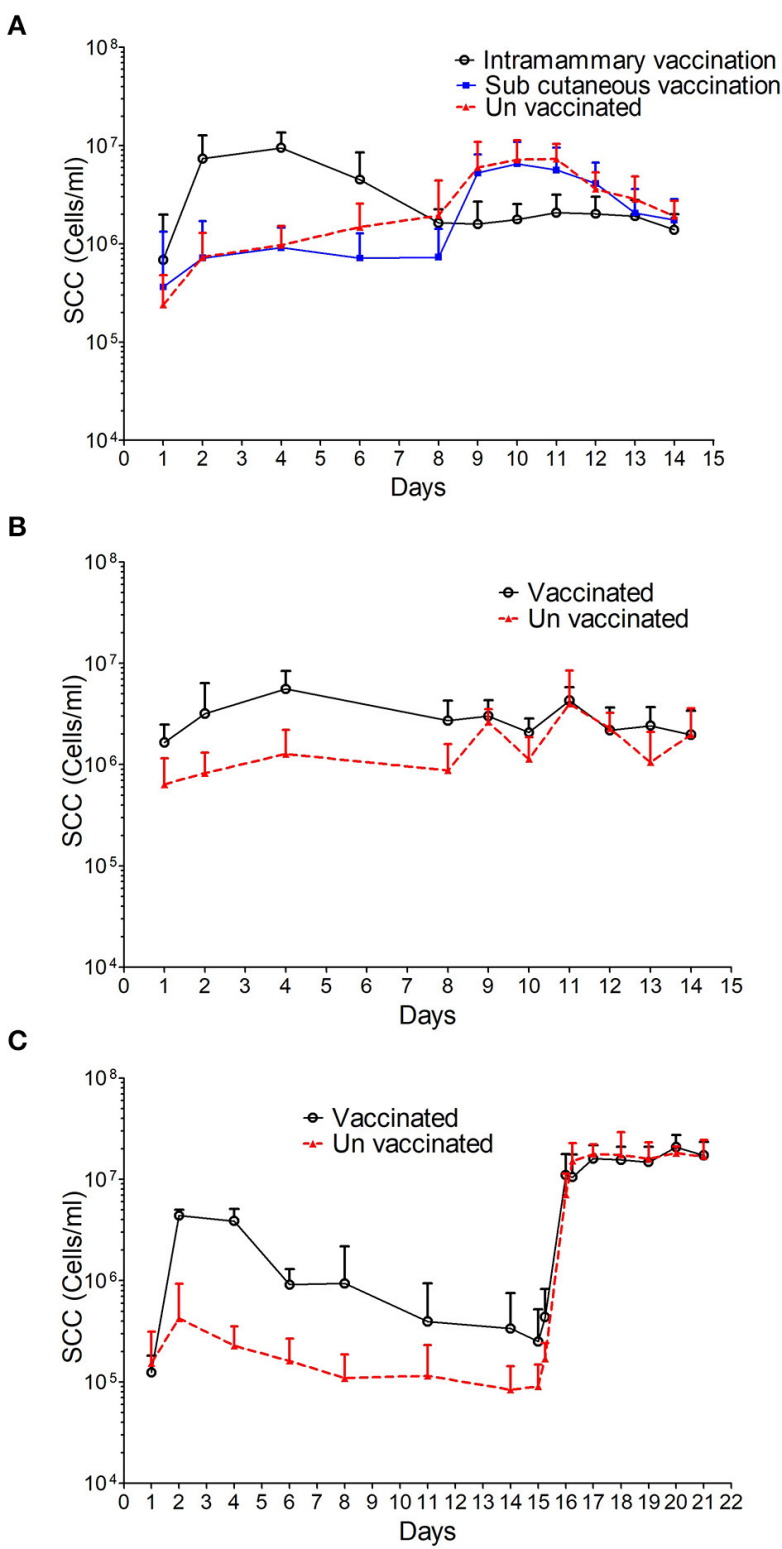
SCC data from sheep immunised and challenged with *M. haemolytica*. The average SCC in cells/ml with standard deviation is shown for study 1 **(A)** where three groups are represented: i, intramammary immunisation; ii, sub-cutaneous immunisation and iii, unvaccinated controls; study 2 **(B)** where two groups are represented: i, intramammary immunisation and ii, un-vaccinated controls and study 3 **(C)** where 2 groups are represented: i, intramammary immunisation and ii, unvaccinated controls.

The SCC increased in all three studies in response to intramammary immunisation compared with the unvaccinated controls. The increase was observed 24 h after immunisation in studies 1 and 2 and reached a peak of 9.49 × 10^6^ ± 4.06 × 10^6^ and 5.57 × 10^6^ ± 2.85 × 10^6^ cells/ml, respectively at 72 h (Figures 3A,B). This represented an increase of 1.4 and 3.8-fold compared with pre-immunisation levels. The SCC remained statistically significantly higher in intramammary immunised animals compared with control and subcutaneously immunised animals until day 7 (*p* < 0.001, Supplementary Figure 1). Prior to challenge at day 8 no statistically significant differences were observed between immunised and control groups (*p* = 0.050).

In study 3 the SCC increased at 24 h post-immunisation in the intramammary group with a peak concentration of 4.39 × 10^6^ ± 6.38 × 10^5^ cells/ml, which was 35-fold higher than the pre-immunisation levels. The SCC decreased thereafter and by day 8 was comparable to that observed in the unvaccinated control group prior to challenge (*p* = 0.184).

No differences in SCC were observed in animals that received the subcutaneous immunisation in study 1 compared to the control animals (Supplementary Figure 1).

The SCC increased in all control animals in response to intramammary challenge (Figures 3A–C). At 24 h post-challenge the mean SSC corresponded to 6.01 × 10^6^ ± 4.88 × 10^6^ and 2.63 × 10^6^ ± 9.02 × 10^5^ cells/ml in studies 1 and 2, respectively, representing a 2-fold increase when compared with pre-challenge levels. In study 3, the SCC changed from 9.06 × 10^4^ ± 5.83 × 10^4^ cells/ml to 7.1 × 10^6^ ± 4.25 × 10^6^ cells/ml representing an 80-fold increase when compared with pre-challenge levels and remained at this level until the end of the study. In studies 1 and 2, a 3 and 1.5-fold decrease, respectively in the mean SCC was observed in control animals at 6 days PC. The SCC in animals subcutaneously immunised in study 1 followed a pattern similar to that observed in control animals with a 7-fold increase in count from 7.34 × 10^5^ ± 6.94 × 10^5^ to 5.28 × 10^6^ ± 2.86 × 10^6^ cells/ml 24 h PC.

In studies 1 and 2, no increase in SCC was observed following challenge in those animals that received an intramammary immunisation. In contrast, the SCC in animals immunised in study 3 followed a pattern similar to that observed in control animals with a 40-fold increase in cell counts from 2.5 × 10^5^ ± 2.71 × 10^5^ to 1.11 × 10^7^ ± 6.75 × 10^6^ cells/ml at 24 h PC (Figures 3A–C). Overall, despite the variability in the SCC after challenge no statistically significant differences were found between vaccinated animals and control animals after the challenge (Supplementary Figure 1).

Cytokine responses in milk following immunisation and intramammary challenge

**Figure 4:**
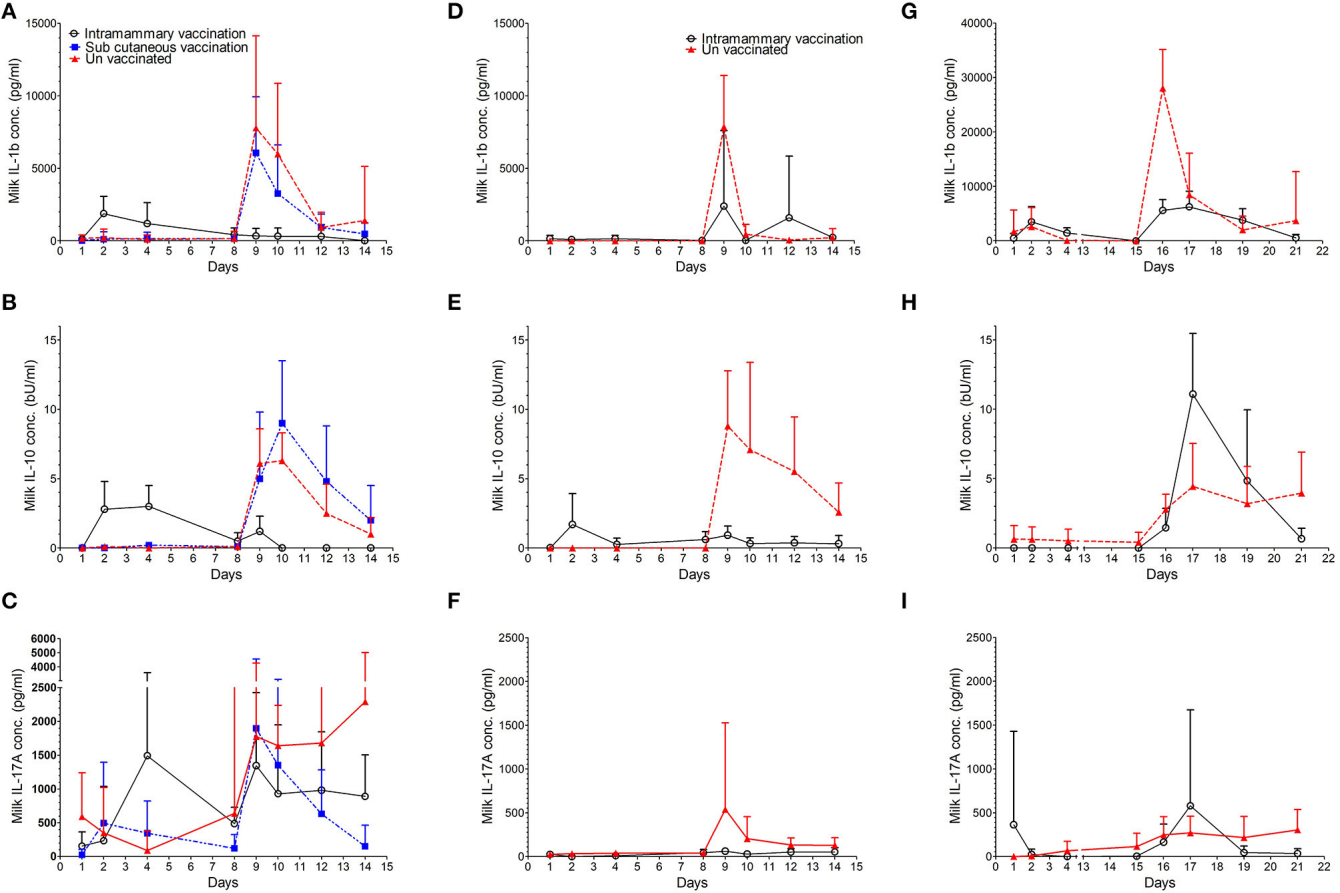
Concentration of cytokines IL-1β, IL-10, and IL-17A in milk. The average concentration with standard deviation is shown for IL-1β in study 1 **(A)**, study 2 **(D)**, and study 3 **(G)**. The average concentration with standard deviation is shown for IL-10 in study 1 **(B)**, study 2 **(E)**, and study 3 **(H)**. The average concentration with standard deviation is shown for IL-17A in study 1 **(C)**, study 2 **(F)**, and study 3 **(I)**.

In order to understand the specific mechanisms behind the protective immune response observed when animals were challenged 7 days post immunisation we measured the concentration of some pro and anti-inflammatory cytokines in milk samples from studies 1 to 3. Changes in IL-1β, IL-10, and IL-17A concentrations are shown in Figure 4 and actual values at key time points are shown in Table 2. The output of the models used is reported in Supplementary Tables 5–10.

**Table 2:** Concentration of milk cytokines above the baseline pre-vaccination, post-vaccination peak, and post-challenge peak.

	**Pre-vaccination conc. (Day1)**	**Post-vaccination peak conc. and peak time (Day)**	**Post-challenge peak conc. and peak time (Day)**
**Interleukin-1β** **(pg/ml)**			
Experiment 1			
Intramammary vaccination	NA	1882 ± 1117 **(2)**	NA
Subcutaneous vaccination	NA	NA	6076 ± 3749 **(9)**
Controls	NA	NA	7788 ± 6139 **(9)**
Experiment 2			
Intramammary vaccination	NA	NA	2401 ± 5204 **(9)**
Controls	NA	NA	7861 ± 3547 **(9)**
Experiment 3			
Intramammary vaccination	486 ± 875	3501 ± 2760 **(2)**	6245 ± 2858 **(17)**
Controls	1715 ± 3927	2589 ± 3504 **(2)**	28058 ± 7088 **(16)**
**Interleukin-10 (bu/ml)**			
Experiment 1			
Intramammary vaccination	NA	3.19 ± 1.31 **(4)**	1.23 ± 1.26 **(9)**
Subcutaneous vaccination	NA	NA	8.67 ± 4.29 **(10)**
Controls	NA	NA	6.27 ± 1.93 **(10)**
Experiment 2			
Intramammary vaccination	NA	1.70 ± 2.23 **(2)**	0.93 ± 0.65 **(9)**
Controls	NA	NA	8.79 ± 3.98 **(9)**
Experiment 3			
Intramammary vaccination	NA	NA	11.09 ± 4.38 **(17)**
Controls	NA	NA	4.44 ± 3.09 **(17)**
**Interleukin-17A (pg/ml)**			
Experiment 1			
Intramammary vaccination	NA	1317 ± 2067 **(4)**	1359 ± 1086 **(9)**
Subcutaneous vaccination	NA	507 ± 790 **(2)**	1913 ± 2326 **(9)**
Controls	629 ± 679	NA	1725 ± 2240 **(14)**
Experiment 2			
Intramammary vaccination	NA	NA	NA
Controls	NA	NA	540 ± 989 **(9)**
Experiment 3			
Intramammary vaccination	364 ± 1066	NA	580 ± 1094 **(17)**
Controls	NA	NA	305 ± 231 **(21)**

*For each experimental group the average concentration, standard deviation and peak day is shown*.

### Interleukin-1β

During the pre-challenge period low levels of IL-1β were detected in milk samples from control and vaccinated animals in all three studies (Table 2). In studies 1 and 3, the concentration of IL-1β increased 14 and 7-fold, respectively at 24 h post intramammary immunisation when compared to pre immunisation levels (Figures 4A,D,G). No increase in IL1β was detected in animals immunised via the intramammary route in study 2. Similarly, no increase in IL-1β was observed in animals which received the subcutaneous immunisation in study 1.

Over the course of all three studies, pre challenge concentrations of IL-1β in immunised animals were not statistically different from control animals (Supplementary Tables 5,6). Following challenge, IL-1β concentrations increased in control animals from all three studies (Figures 4A,D,G and Table 2). Statistically significant lower concentrations of IL-1β were observed in intramammary immunised animals when compared to controls 24 h after challenge (*p* < 0.001 in studies 1 and 2 and *p* = 0.024 in study 3). In study 3, a further increase of IL-1β was observed in control animals at day 6 when compared to animals that received the intramammary vaccination (*p* = 0.034).

### Interleukin-10

During the pre-challenge period low levels of IL-10 were detected in milk samples from control and vaccinated animals in all three studies (Supplementary Tables 7, 8). IL-10 concentrations increased from 24 h post intramammary immunisation in studies 1 and 2 (Figures 4B,E,H and Table 2) peaking at 72 h post immunisation in animals intramammary vaccinated (Table 2). In these experiments no increase of IL-10 was observed following subcutaneous immunisation or in control animals. No increase of IL-10 following vaccination was observed in experiment 3 in either the vaccinated or the control animals.

In experiments 1 and 2 this resulted in a higher level of IL-10 in animals that received the intramammary immunisation compared with the control animals (*p* < 0.001). The concentration of IL-10 in study 3 could be assessed only post challenge due to model fitting issues stemming from a high proportion of zero responses pre-challenge.

Following challenge, an increase in IL-10 was observed in control animals from all three studies. This resulted in statistically significant lower levels of IL-10 in intramammary immunised animals compared to controls at 24 h (*p* < 0.001), 48 h (*p* < 0.001), and 72 h (*p* = 0.008) PC (Figures 4D–F) in studies 1 and 2. In study 3, however, an increased IL-10 concentration compared to the controls was observed in intramammary immunised animals 48 h PC (*p* = 0.046). This was reversed 6 days post-challenge when control animals had a statistically significantly higher concentration of this cytokine compared to the intramammary immunised animals (*p* = 0.046).

### Interleukin-17A

During the pre-challenge period low levels of IL-17A were detected in milk samples from control and vaccinated animals in all three studies (Supplementary Tables 9, 10). IL-17A concentration increased in study 1, 24 h following intramammary immunisation. Levels peaked at 72 h representing a 10-fold increase compared with pre-immunisation levels (Table 2). In animals which received a subcutaneous immunisation, an 18-fold increase in IL-17A was observed 24 h post immunisation (Figure 4C; Table 2). No increase of IL-17A levels was observed in intramammary vaccinated animals in studies 2 and 3. No increase of IL-17A was observed during the pre-challenge period in control animals in the three experiments.

Despite this variability, there were no statistically significant differences in the pre challenge levels of IL-17A between the vaccinated and the control animals in the three experiments (Supplementary Tables 9, 10). Following challenge, IL-17A levels increased in the control animals from all three studies. The increase was detected 24 h PC which coincided with the maximal levels in study 2 (Figure 4F and Table 2). In studies 1 and 3 IL-17A stayed at elevated levels until the end of the study (Figures 4C,I). This resulted in a statistically significant higher concentration compared to the intramammary immunised animals. This difference was detected in studies 1 and 2, 24 h PC (*p* = 0.008), 48 h PC (*p* = 0.015), 96 h PC (*p* = 0.018), and 144 h PC (*p* = 0.008). In study 3, however, the increase of IL-17A in control animals compared to the intramammary immunised (*p* < 0.001) animals was observed only 6 days PC (*p* = 0.036). No differences in the concentration of the animals vaccinated subcutaneously was observed in comparison to the control animals (Supplementary Table 9).

## Discussion

The aim of this study was to test whether local administration of a vaccine, originally developed to protect sheep against respiratory disease caused by *M. haemolytica*, would also protect against an IMI caused by the same bacterial species. We initially tested two different administration routes; subcutaneous in an area located close to the supramammary draining lymph nodes and intramammary, which consisted of infusion of the vaccine directly into the mammary gland through the teat orifice. The results clearly demonstrated that intramammary immunisation protected against subsequent bacterial challenge with *M. haemolytica*. In two independent studies, animals which received the vaccine through the intramammary route did not develop intramammary infections following challenge. Over these studies, only 2 of the 24 mammary halves that were immunised tested positive for bacteria 1 week post challenged. In comparison, 28 out of the 30 control mammary halves developed intramammary infections. However, the protective effect observed at 7 days was not observed when animals were challenged 14 days after intramammary immunisation. In this study, immunisation failed to reduce infection, bacterial concentration in the milk or impact the SCC when compared with controls. Similarly, no protection against challenge was observed in animals immunised through the subcutaneous route close to the supramammary draining lymph nodes although a five-fold reduction in bacterial load was observed compared to the control animals, this was not statistically different to that observed in unvaccinated control animals.

In our intramammary challenge model, infected glands showed clinical signs of mastitis such as redness, pain on palpation and the presence of clots and discoloration of the milk within 24 h. This is in accordance with the experimental models previously described in the literature (32, 33). Bacteria could be recovered from the milk by 12 h PC and reached a peak 24 h PC. The bacterial concentration decreased thereafter and many animals progressively cleared the infection. Bacterial clearance appears to be related to an increase in SCC and is likely to include neutrophils and macrophages which are known to play a bactericidal role (34). A similar infection pattern has been described for other Gram-negative mastitis pathogens in cattle such as *E. coli, Serratia marcescens*, and *Klebsiella pneumoniae* (35).

The delivery of vaccine antigens directly into the mammary gland through the teat has previously been investigated as a means of protecting against IMI in ruminant livestock. Both antibody and cellular immune responses to immunogens such as tuberculin (36) and ovalbumin (37) may be induced by delivery of antigens directly into the mammary gland. Importantly for vaccine development, the induction of immune memory through delivery of vaccine antigens directly into the mammary gland has also been demonstrated (36). These observations were further expanded in studies that applied the intramammary delivery route to important mastitis pathogens (19). Studies conducted mainly in cattle with either whole killed bacteria or bacterial antigens demonstrated that delivery directly into the mammary gland elicited an enhanced response compared to the same vaccine delivered through a subcutaneous or an intramuscular injection. For example, Finch et al. (23) demonstrated that intramammary immunisation with killed *S. uberis* during the dry period induces a protective response to homologous challenge. In the same study animals were immunised subcutaneously with the same vaccine preparation but with the addition of an adjuvant. The addition of an adjuvant reduced the number of bacteria in the challenged mammary glands whereas intramammary vaccinated animals did not. Protection did not appear to correlate with milk antibody titre as both subcutaneous and intramammary immunisations showed similar concentration of antibodies.

In contrast to studies carried out in dairy cattle where intramammary immunisation was administered during the dry period, we have focused on sheep during lactation. For these reasons care should be taken when making direct comparisons between studies. Nevertheless, the protective effect of intramammary immunisation described here is similar to related studies conducted in cattle. The protective effect of the intramammary immunisation protocol we tested appears to be limited to 7 days. It is possible that the duration of protection may be extended by employing multiple administrations of the vaccine, different adjuvants and combinations of intramammary and subcutaneous immunisation regimes. Also in the studies described here immunologically naïve animals were not available as pregnant ewes are immunised subcutaneously with Ovipast or similar vaccines, prior to parturition as part of routine flock management in order to protect against respiratory disease. Our immunisation strategy may therefore be considered a systemic prime followed by an intramammary boost.

It was surprising therefore that boosting the immune response through re-stimulation of previously primed sheep via intramammary administration of vaccine antigens was not mirrored by subcutaneous administration over the supramammary lymph nodes. It is possible that the limited effect on bacterial numbers following subcutaneous immunisation reflects a boosting of an acquired immune response. On the other hand an intramammary boost may have primed innate immune effectors cells such as neutrophils and macrophages as well as boosting the antibody responses. Future cellular and serological analyses will seek to confirm this.

In our study, intramammary immunisation induced an inflammatory response within the mammary gland, characterised by increases in SCC (Figure 3) and in the production of cytokines IL-1β, IL-10 and IL-17A (Figure 4). The SCC and cytokine levels appeared to have returned to levels comparable with un-immunised controls prior to challenge except for IL-10 in animals challenged 7 days after immunisation. This raises a number of questions regarding the immune mechanisms associated with protection at 7 days and why it is greatly reduced at 14 days post-immunisation. The protective effect may be due to boosting and targeting to the mammary gland of an adaptive immune response which includes immunoglobulin and cell mediated responses. However, the reduced levels of protection observed at 14 days when we would expect adaptive responses to remain effective, suggests that mechanisms other than adaptive immunity may be involved. Rainard et al. (38) found that infusion of inflammatory compounds such as lipopolysaccharides (LPS) and ovalbumin into the mammary gland induced an inflammatory response that lasted no longer than 72 h. Our immunisation strategy may have induced similar innate responses within the mammary gland which remained effective at 7 days post-immunisation but substantially reduced at 14 days.

Intramammary immunisation with gram negative bacterial cell wall components such as LPS (39) also produced results on challenge similar to those we describe here. In addition to bacterial antigens that induce protective responses against respiratory infection, the Ovipast vaccine is likely to also include bacterial components that act as ligands for pathogen recognition receptors including the Toll-like receptors (TLR). TLR are expressed by mammary epithelial cells (40) which in *in vitro* and *in vivo* studies rapidly respond to bacterial antigens including LPS (41). Activation of the mammary epithelium results in the production of antibacterial peptides (42), as well as cytokines and chemokines which recruit macrophages and neutrophils into the mammary gland (43). The rapid and almost sterile protection we observed in study 1 indicates that the challenge was effectively eliminated upon delivery to the mammary gland. Whether this was mediated by such innate mechanisms or through a combination of innate and adaptive immune mechanisms will be determined in future studies using this model.

To begin the investigation of the nature of the immune response in the ovine mammary gland following immunisation and challenge we measured the concentration of IL-1β, IL-10, and IL-17A in milk samples over the course of each study. The pro inflammatory cytokine IL-1β closely mirrors the SCC reaching its peak 24 h PC and declining thereafter. This cytokine is produced in response to pathogens by both macrophages (44) and mammary epithelial cells (45) and plays a fundamental role in initiating the inflammatory response in the mammary gland (35). In this study, the early detection of this cytokine is consistent with such a role. The peak of IL-1β is followed by IL-10 which is mainly produced by macrophages and lymphocytes. IL-10 has an anti-inflammatory role which is crucial in down regulating inflammation in order to avoid excessive tissue damage. A similar pattern of pro- and anti-inflammatory cytokine production has been described for several cattle mastitis pathogens (35).

We also detected IL-17A in response to infection although the cellular source of this cytokine and its role in protection are not clear. The protection observed at 7 days following intramammary immunisation was not related to an increase in IL-17A in milk either after vaccination or in response to the challenge. This suggest that IL-17A may not be produced by Th17 lymphocytes as part of an adaptive immune response to challenge. It may however be produced by a range of innate immune cells which have central roles in the maintenance of mucosal immunity (46). Notwithstanding it source, IL-17 is considered important in the response to extra cellular pathogens as it has been shown to recruit and enhance the bactericidal activity of phagocytes (47). *In vitro*, IL-17 family cytokines up regulate the production of antimicrobial proteins from bovine mammary epithelial cells (22), which contributes to bacterial clearance. We have previously reported that induction of IL-17A in cattle infected with *S. uberis* appeared to be associated with neutrophil recruitment and clearance of bacterial infection (20). The detection of IL-17A in multiple hosts and in response to different pathogen species confirms its importance in the context of mastitis and suggests that its induction through innate and or adaptive immune stimulation may be important in protecting against IMI.

In conclusion, we have demonstrated that delivery of a vaccine directly into the mammary gland via the teat canal can induce a protective response against subsequent IMI. While the protective effect was limited in duration it provides us with a cost effective, small ruminant immunisation, and challenge model to study the induction, nature and manipulation of immunity within the ruminant mammary gland. Future studies will seek to determine the relative importance of innate and adaptive responses in the protection observed 7 days post-immunisation and whether these may ultimately be exploited to improve ruminant udder health.

## Data Availability Statement

The original contributions presented in the study are included in the article/Supplementary Material, further inquiries can be directed to the corresponding author/s.

## Ethics Statement

The animal study was reviewed and approved by Moredun Research Institute Animal Welfare and Ethical Review Body.

## Author Contributions

RT and KB conceived and designed the experiments. RT, JF, MS, HT, and KB performed the experiments. DE performed the statistical analysis. RT, DE, and KB wrote the manuscript. All authors contributed to the article and approved the submitted version.

## Conflict of Interest

The authors declare that the research was conducted in the absence of any commercial or financial relationships that could be construed as a potential conflict of interest.
