# Supplementary material for: Intramammary Immunisation Provides Short Term Protection Against Mannheimia haemolytica Mastitis in Sheep
Source: Front Vet Sci. 2021 Jun 10;8:659803. doi: 10.3389/fvets.2021.659803 (PMC8222732; doi:10.3389/fvets.2021.659803)
Supplement: Supplementary file 1 [file Data_Sheet_1.docx]

Supplementary Material

|  | **Bacteria transf** | | |  |
| --- | --- | --- | --- | --- |
| *Predictors* | *Estimates* | *CI* | *p* |  |
| **Count Model** | | | | |
| (Intercept) | 7.92 | 6.14 – 9.71 | **<0.001** |  |
| Transformed.Time | -3.54 | -4.52 – -2.57 | **<0.001** |  |
| Transformed.Time^2 | 0.52 | 0.32 – 0.72 | **<0.001** |  |
| **Zero-Inflated Model** | | | |  |
| (Intercept) | -2.04 | -3.70 – -0.38 | **0.016** |  |
| Treatment [SC] | -1.02 | -3.44 – 1.40 | 0.409 |  |
| Treatment [IMM] | 6.84 | 3.58 – 10.11 | **<0.001** |  |
| Transformed.Time | 0.76 | 0.50 – 1.02 | **<0.001** |  |
| **Random Effects** | | | |  |
| σ^2^ | 9.27 | | |  |
| τ_00_ _Animal:Experiment_ | 2.39 | | |  |
| τ_00_ _Experiment_ | 0.91 | | |  |
| ICC | 0.26 | | |  |
| N _Animal_ | 35 | | |  |
| N _Experiment_ | 2 | | |  |
| Observations | 418 | | |  |
| Marginal R^2^ / Conditional R^2^ | 0.254 / 0.450 | | |  |

Table 1. Bacteria in milk. Study 1 and 2

|  | **Bacteria transf** | | |  |
| --- | --- | --- | --- | --- |
| *Predictors* | *Estimates* | *CI* | *p* |  |
| **Count Model** | | | | |
| (Intercept) | 14.01 | 12.53 – 15.49 | **<0.001** |  |
| Transformed.Time | -8.26 | -9.54 – -6.99 | **<0.001** |  |
| Transformed.Time^2 | 1.27 | 1.01 – 1.54 | **<0.001** |  |
| **Zero-Inflated Model** | | | |  |
| (Intercept) | -5.13 | -7.87 – -2.40 | **<0.001** |  |
| Transformed.Time | 1.41 | 0.77 – 2.06 | **<0.001** |  |
| **Random Effects** | | | |  |
| σ^2^ | 7.70 | | |  |
| τ_00_ _Animal_ | 2.12 | | |  |
| ICC | 0.22 | | |  |
| N _Animal_ | 10 | | |  |
| Observations | 117 | | |  |
| Marginal R^2^ / Conditional R^2^ | 0.682 / 0.751 | | |  |

Table 2. Bacteria in milk. Study 3

|  | **log SCC** | |  |
| --- | --- | --- | --- |
| *Predictors* | *Estimates* | *CI* | *p* |
| (Intercept) | 14.16 | 13.98 – 14.34 | **<0.001** |
| TreatmentSC | -0.16 | -0.43 – 0.12 | 0.264 |
| TreatmentIMM | 0.33 | 0.06 – 0.60 | **0.016** |
| Smooth term (Time) : TreatmentNO | 8.97 |  | **<0.001** |
| Smooth term (Time) : TreatmentSC | 8.69 |  | **<0.001** |
| Smooth term (Time) : TreatmentIMM | 7.61 |  | **<0.001** |
| Smooth term (Time,Animal) | 48.00 |  | **<0.001** |
| Observations | 730 | | |
| R^2^ | 0.621 | | |

Table 3 Somatic cell count Studies 1 and 2

| *contrast* | *estimate* | *SE* | *df* | *t.ratio* | *p.value* |
| --- | --- | --- | --- | --- | --- |
| NO - SC | 0.434 | 0.371 | 31.000 | 1.170 | 0.251 |
| NO - IMM | -0.804 | 0.361 | 31.000 | -2.226 | 0.050 |
| SC - IMM | -1.238 | 0.385 | 31.000 | -3.213 | 0.009 |

Table 4 Somatic cell count Study 3

|  | | | **log SCC** | | | | |
| --- | --- | --- | --- | --- | --- | --- | --- |
| *Predictors* | | | *Estimates* | | *CI* | | *p* |
| (Intercept) | | | 13.55 | | 12.31 – 14.80 | | **<0.001** |
| TreatmentIMM | | | 0.98 | | -0.79 – 2.74 | | 0.278 |
| Smooth term (Time) : TreatmentNO | | | 12.41 | |  | | **<0.001** |
| Smooth term (Time) : TreatmentIMM | | | 12.54 | |  | | **<0.001** |
| Smooth term (Time,Animal) | | | 27.59 | |  | | **<0.001** |
| Observations | | | 269 | | | | |
| R^2^ | | | 0.951 | | | | |
| *contrast* | *estimate* | *SE* | | *df* | *t.ratio* | *p.value* |  |
| NO - IMM | -0.715 | 0.492 | | 8.000 | -1.454 | 0.184 |  |

Table 5 Interleukin 1β studies 1 and 2

| **log IL 1 b** | | | | |  |  |
| --- | --- | --- | --- | --- | --- | --- |
| *Predictors* | | *Estimates* | | *CI* | *p* |  |
| **Count Model** | | | | | | |
| (Intercept) | | 5.34 | | 4.11 – 6.58 | **<0.001** |  |
| Time.Group [Day 9] | | 3.14 | | 2.25 – 4.04 | **<0.001** |  |
| Time.Group [Day 10] | | 1.78 | | 0.67 – 2.89 | **0.002** |  |
| Time.Group [Day 12] | | 0.53 | | -0.50 – 1.56 | 0.316 |  |
| Time.Group [Day 14] | | -0.08 | | -1.14 – 0.98 | 0.882 |  |
| Treatment [SC] | | -0.69 | | -1.72 – 0.34 | 0.188 |  |
| Treatment [IMM] | | 0.12 | | -0.70 – 0.94 | 0.780 |  |
| Time.Group [Day 9] * Treatment [SC] | | 0.20 | | -1.14 – 1.54 | 0.772 |  |
| Time.Group [Day 10] * Treatment [SC] | | 0.79 | | -0.73 – 2.31 | 0.307 |  |
| Time.Group [Day 12] * Treatment [SC] | | 1.50 | | -0.02 – 3.02 | 0.052 |  |
| Time.Group [Day 14] * Treatment [SC] | | 1.53 | | -0.06 – 3.12 | 0.059 |  |
| Time.Group [Day 9] * Treatment [IMM] | | -3.14 | | -4.39 – -1.89 | **<0.001** |  |
| Time.Group [Day 10] * Treatment [IMM] | | -1.66 | | -3.19 – -0.13 | **0.034** |  |
| Time.Group [Day 12] * Treatment [IMM] | | 0.33 | | -1.45 – 2.11 | 0.715 |  |
| Time.Group [Day 14] * Treatment [IMM] | | -0.80 | | -2.49 – 0.90 | 0.357 |  |
| **Zero-Inflated Model** | | | | | |  |
| (Intercept) | | 0.71 | | 0.32 – 1.11 | **<0.001** |  |
| Time.Group [Day 9] | | -2.73 | | -3.54 – -1.91 | **<0.001** |  |
| Time.Group [Day 10] | | -1.46 | | -2.16 – -0.77 | **<0.001** |  |
| Time.Group [Day 12] | | -0.55 | | -1.16 – 0.05 | 0.073 |  |
| Time.Group [Day 14] | | -0.40 | | -1.01 – 0.22 | 0.207 |  |
| **Random Effects** | | | | | |  |
| σ^2^ | | 1.98 | | | |  |
| τ_00_ _Animal:Experiment_ | | 0.08 | | | |  |
| τ_00_ _Experiment_ | | 0.51 | | | |  |
| ICC | | 0.23 | | | |  |
| N _Animal_ | | 35 | | | |  |
| N _Experiment_ | | 2 | | | |  |
| Observations | | 494 | | | |  |
| Marginal R^2^ / Conditional R^2^ | | 0.292 / 0.455 | | | |  |
| *contrast* | *Time.Group* | *estimate* | *SE* | *df* | *t.ratio* | *p.value* |
| NO - SC | Pre-challenge | 0.691 | 0.525 | 485 | 1.317 | 0.322 |
| NO - IMM | Pre-challenge | -0.117 | 0.418 | 485 | -0.279 | 0.836 |
| SC - IMM | Pre-challenge | -0.808 | 0.449 | 485 | -1.797 | 0.182 |
| NO - SC | Day 9 | 0.493 | 0.474 | 485 | 1.041 | 0.448 |
| NO - IMM | Day 9 | 3.019 | 0.512 | 485 | 5.892 | 0.000 |
| SC - IMM | Day 9 | 2.526 | 0.563 | 485 | 4.485 | 0.000 |
| NO - SC | Day 10 | -0.100 | 0.594 | 485 | -0.169 | 0.866 |
| NO - IMM | Day 10 | 1.542 | 0.680 | 485 | 2.270 | 0.089 |
| SC - IMM | Day 10 | 1.643 | 0.650 | 485 | 2.528 | 0.059 |
| NO - SC | Day 12 | -0.811 | 0.596 | 485 | -1.361 | 0.322 |
| NO - IMM | Day 12 | -0.448 | 0.827 | 485 | -0.542 | 0.735 |
| SC - IMM | Day 12 | 0.363 | 0.858 | 485 | 0.423 | 0.776 |
| NO - SC | Day 14 | -0.839 | 0.644 | 485 | -1.303 | 0.322 |
| NO - IMM | Day 14 | 0.679 | 0.782 | 485 | 0.869 | 0.526 |
| SC - IMM | Day 14 | 1.518 | 0.827 | 485 | 1.835 | 0.182 |

Table 6 Interleukin 1β study 3

|  | | **log IL 1 b** | | | | |  |
| --- | --- | --- | --- | --- | --- | --- | --- |
| *Predictors* | | *Estimates* | | *CI* | | *p* |  |
| **Count Model** | | | | | | | |
| (Intercept) | | 7.13 | | 6.41 – 7.85 | | **<0.001** |  |
| Time.Group [Day 16] | | 3.08 | | 2.11 – 4.06 | | **<0.001** |  |
| Time.Group [Day 17] | | 1.79 | | 0.79 – 2.80 | | **<0.001** |  |
| Time.Group [Day 19] | | 0.57 | | -0.52 – 1.66 | | 0.307 |  |
| Time.Group [Day 21] | | 0.32 | | -0.82 – 1.46 | | 0.582 |  |
| Treatment [IMM] | | -0.29 | | -1.20 – 0.61 | | 0.524 |  |
| Time.Group [Day 16] * Treatment [IMM] | | -1.35 | | -2.64 – -0.05 | | **0.042** |  |
| Time.Group [Day 17] * Treatment [IMM] | | 0.02 | | -1.30 – 1.34 | | 0.980 |  |
| Time.Group [Day 19] * Treatment [IMM] | | 0.49 | | -0.89 – 1.88 | | 0.485 |  |
| Time.Group [Day 21] * Treatment [IMM] | | -1.39 | | -2.86 – 0.08 | | 0.063 |  |
| **Zero-Inflated Model** | | | | | | |  |
| (Intercept) | | 0.25 | | -0.74 – 1.23 | | 0.625 |  |
| Treatment [IMM] | | -2.25 | | -3.77 – -0.73 | | **0.004** |  |
| Time.Group [Day 16] | | -20.18 | | -11144.73 – 11104.37 | | 0.997 |  |
| Time.Group [Day 17] | | -2.78 | | -4.98 – -0.57 | | **0.014** |  |
| Time.Group [Day 19] | | -1.42 | | -2.93 – 0.08 | | 0.063 |  |
| Time.Group [Day 21] | | -0.31 | | -1.58 – 0.96 | | 0.634 |  |
| **Random Effects** | | | | | | |  |
| σ^2^ | | 1.38 | | | | |  |
| τ_00_ _Animal_ | | 0.13 | | | | |  |
| ICC | | 0.08 | | | | |  |
| N _Animal_ | | 10 | | | | |  |
| Observations | | 140 | | | | |  |
| Marginal R^2^ / Conditional R^2^ | | 0.441 / 0.488 | | | | |  |
| *contrast* | *Time.Group* | *estimate* | *SE* | *df* | *t.ratio* | *p.value* |  |
| NO - IMM | Pre-challenge | 0.294 | 0.462 | 131 | 0.637 | 0.748 |  |
| NO - IMM | Day 16 | 1.640 | 0.571 | 131 | 2.874 | 0.024 |  |
| NO - IMM | Day 17 | 0.278 | 0.585 | 131 | 0.475 | 0.748 |  |
| NO - IMM | Day 19 | -0.200 | 0.622 | 131 | -0.322 | 0.748 |  |
| NO - IMM | Day 21 | 1.688 | 0.677 | 131 | 2.493 | 0.035 |  |

Table 7 Interleukin 10 studies 1 and 2

|  | | **IL 10** | | | |  |
| --- | --- | --- | --- | --- | --- | --- |
| *Predictors* | | *Estimates* | | *CI* | *p* |  |
| **Count Model** | | | | | | |
| (Intercept) | | -3.20 | | -3.79 – -2.61 | **<0.001** |  |
| Time.Group [Day 9] | | 5.16 | | 4.55 – 5.77 | **<0.001** |  |
| Time.Group [Day 10] | | 5.06 | | 4.41 – 5.71 | **<0.001** |  |
| Time.Group [Day 12] | | 4.68 | | 4.03 – 5.32 | **<0.001** |  |
| Time.Group [Day 14] | | 3.95 | | 3.26 – 4.65 | **<0.001** |  |
| Treatment [SC] | | 0.94 | | 0.17 – 1.72 | **0.017** |  |
| Treatment [IMM] | | 4.01 | | 3.34 – 4.67 | **<0.001** |  |
| Time.Group [Day 9] * Treatment [SC] | | -1.21 | | -2.04 – -0.39 | **0.004** |  |
| Time.Group [Day 10] * Treatment [SC] | | -0.65 | | -1.49 – 0.18 | 0.126 |  |
| Time.Group [Day 12] * Treatment [SC] | | -0.74 | | -1.59 – 0.12 | 0.090 |  |
| Time.Group [Day 14] * Treatment [SC] | | -0.63 | | -1.57 – 0.32 | 0.193 |  |
| Time.Group [Day 9] * Treatment [IMM] | | -5.73 | | -6.55 – -4.91 | **<0.001** |  |
| Time.Group [Day 10] * Treatment [IMM] | | -6.90 | | -8.55 – -5.24 | **<0.001** |  |
| Time.Group [Day 12] * Treatment [IMM] | | -5.94 | | -7.40 – -4.48 | **<0.001** |  |
| Time.Group [Day 14] * Treatment [IMM] | | -4.68 | | -6.39 – -2.97 | **<0.001** |  |
| **Zero-Inflated Model** | | | | | |  |
| (Intercept) | | -3.67 | | -5.76 – -1.57 | **0.001** |  |
| Treatment [SC] | | -0.18 | | -1.68 – 1.32 | 0.815 |  |
| Treatment [IMM] | | 3.36 | | 1.51 – 5.21 | **<0.001** |  |
| Time.Group [Day 9] | | -1.53 | | -3.83 – 0.76 | 0.191 |  |
| Time.Group [Day 10] | | 0.71 | | -1.12 – 2.54 | 0.447 |  |
| Time.Group [Day 12] | | 1.69 | | 0.10 – 3.28 | **0.037** |  |
| Time.Group [Day 14] | | 2.40 | | 0.70 – 4.11 | **0.006** |  |
| **Random Effects** | | | | | |  |
| σ^2^ | | 0.39 | | | |  |
| τ_00_ _Animal:Experiment_ | | 0.06 | | | |  |
| τ_00_ _Experiment_ | | 0.00 | | | |  |
| N _Animal_ | | 35 | | | |  |
| N _Experiment_ | | 2 | | | |  |
| Observations | | 498 | | | |  |
| Marginal R^2^ / Conditional R^2^ | | 0.905 / NA | | | |  |
| *contrast* | *Time.Group* | *estimate* | *SE* | *df* | *t.ratio* | *p.value* |
| NO - SC | Pre-challenge | -0.941 | 0.395 | 486 | -2.383 | 0.029 |
| NO - IMM | Pre-challenge | -4.006 | 0.338 | 486 | -11.869 | 0.000 |
| SC - IMM | Pre-challenge | -3.065 | 0.302 | 486 | -10.136 | 0.000 |
| NO - SC | Day 9 | 0.273 | 0.204 | 486 | 1.341 | 0.256 |
| NO - IMM | Day 9 | 1.723 | 0.284 | 486 | 6.066 | 0.000 |
| SC - IMM | Day 9 | 1.450 | 0.297 | 486 | 4.880 | 0.000 |
| NO - SC | Day 10 | -0.286 | 0.217 | 486 | -1.318 | 0.256 |
| NO - IMM | Day 10 | 2.893 | 0.780 | 486 | 3.709 | 0.001 |
| SC - IMM | Day 10 | 3.179 | 0.772 | 486 | 4.116 | 0.000 |
| NO - SC | Day 12 | -0.202 | 0.234 | 486 | -0.864 | 0.408 |
| NO - IMM | Day 12 | 1.938 | 0.672 | 486 | 2.883 | 0.008 |
| SC - IMM | Day 12 | 2.140 | 0.671 | 486 | 3.192 | 0.003 |
| NO - SC | Day 14 | -0.316 | 0.310 | 486 | -1.020 | 0.356 |
| NO - IMM | Day 14 | 0.673 | 0.812 | 486 | 0.828 | 0.408 |
| SC - IMM | Day 14 | 0.989 | 0.815 | 486 | 1.214 | 0.282 |

Table 8 Interleukin 10 study 3

|  | | **log(IL 10+1)** | | | | |
| --- | --- | --- | --- | --- | --- | --- |
| *Predictors* | | *Estimates* | | *CI* | *p* | |
| (Intercept) | | 1.29 | | 0.76 – 1.82 | **<0.001** | |
| Time.Group [Day 17] | | 0.13 | | -0.36 – 0.62 | 0.602 | |
| Time.Group [Day 19] | | -0.09 | | -0.58 – 0.40 | 0.715 | |
| Time.Group [Day 21] | | 0.07 | | -0.41 – 0.56 | 0.767 | |
| Treatment [IMM] | | -0.57 | | -1.32 – 0.18 | 0.137 | |
| Time.Group [Day 17] * Treatment [IMM] | | 1.58 | | 0.89 – 2.27 | **<0.001** | |
| Time.Group [Day 19] * Treatment [IMM] | | 0.66 | | -0.03 – 1.35 | 0.060 | |
| Time.Group [Day 21] * Treatment [IMM] | | -0.39 | | -1.07 – 0.30 | 0.272 | |
| **Random Effects** | | | | | | |
| σ^2^ | | 0.31 | | | | |
| τ_00_ _Animal_ | | 0.21 | | | | |
| ICC | | 0.40 | | | | |
| N _Animal_ | | 10 | | | | |
| Observations | | 80 | | | | |
| Marginal R^2^ / Conditional R^2^ | | 0.372 / 0.626 | | | | |
| *contrast* | *Time.Group* | *estimate* | *SE* | *df* | *t.ratio* | *p.value* |
| NO - IMM | Day 16 | 0.567 | 0.382 | 16.757 | 1.487 | 0.208 |
| NO - IMM | Day 17 | -1.011 | 0.382 | 16.757 | -2.650 | 0.046 |
| NO - IMM | Day 19 | -0.095 | 0.382 | 16.757 | -0.249 | 0.806 |
| NO - IMM | Day 21 | 0.953 | 0.382 | 16.757 | 2.498 | 0.046 |

Table 9 Interleukin 17A studies 1 and 2

|  | | **log IL 17** | | | | |  |
| --- | --- | --- | --- | --- | --- | --- | --- |
| *Predictors* | | *Estimates* | | *CI* | | *p* |  |
| **Count Model** | | | | | | | |
| (Intercept) | | 5.35 | | 2.55 – 8.15 | | **<0.001** |  |
| Time.Group [Day 9] | | 0.99 | | 0.58 – 1.39 | | **<0.001** |  |
| Time.Group [Day 10] | | 0.62 | | 0.11 – 1.12 | | **0.017** |  |
| Time.Group [Day 12] | | 0.56 | | 0.15 – 0.97 | | **0.007** |  |
| Time.Group [Day 14] | | 0.75 | | 0.32 – 1.18 | | **0.001** |  |
| Treatment [SC] | | -0.84 | | -1.37 – -0.31 | | **0.002** |  |
| Treatment [IMM] | | -0.39 | | -0.89 – 0.10 | | 0.119 |  |
| Time.Group [Day 9] * Treatment [SC] | | 0.43 | | -0.21 – 1.07 | | 0.186 |  |
| Time.Group [Day 10] * Treatment [SC] | | 0.77 | | 0.04 – 1.51 | | **0.039** |  |
| Time.Group [Day 12] * Treatment [SC] | | 0.47 | | -0.21 – 1.16 | | 0.177 |  |
| Time.Group [Day 14] * Treatment [SC] | | -0.86 | | -1.68 – -0.04 | | **0.041** |  |
| Time.Group [Day 9] * Treatment [IMM] | | -0.56 | | -1.16 – 0.04 | | 0.069 |  |
| Time.Group [Day 10] * Treatment [IMM] | | -0.57 | | -1.26 – 0.11 | | 0.099 |  |
| Time.Group [Day 12] * Treatment [IMM] | | -0.41 | | -1.01 – 0.19 | | 0.182 |  |
| Time.Group [Day 14] * Treatment [IMM] | | -0.59 | | -1.20 – 0.03 | | 0.061 |  |
| **Zero-Inflated Model** | | | | | | |  |
| (Intercept) | | 0.13 | | -0.74 – 1.00 | | 0.769 |  |
| Treatment [SC] | | -0.02 | | -1.13 – 1.10 | | 0.976 |  |
| Treatment [IMM] | | -0.00 | | -1.01 – 1.01 | | 0.996 |  |
| Time.Group [Day 9] | | -2.19 | | -3.41 – -0.97 | | **<0.001** |  |
| Time.Group [Day 10] | | -2.18 | | -3.84 – -0.52 | | **0.010** |  |
| Time.Group [Day 12] | | -3.77 | | -5.88 – -1.66 | | **<0.001** |  |
| Time.Group [Day 14] | | -2.82 | | -4.43 – -1.21 | | **0.001** |  |
| Treatment [SC] * Time.Group [Day 9] | | 0.28 | | -1.49 – 2.04 | | 0.759 |  |
| Treatment [IMM] * Time.Group [Day 9] | | -17.55 | | -7731.12 – 7696.03 | | 0.996 |  |
| Treatment [SC] * Time.Group [Day 10] | | 0.64 | | -1.42 – 2.70 | | 0.545 |  |
| Treatment [IMM] * Time.Group [Day 10] | | 0.51 | | -1.56 – 2.58 | | 0.629 |  |
| Treatment [SC] * Time.Group [Day 12] | | 2.81 | | 0.41 – 5.21 | | **0.022** |  |
| Treatment [IMM] * Time.Group [Day 12] | | 0.81 | | -1.89 – 3.52 | | 0.556 |  |
| Treatment [SC] * Time.Group [Day 14] | | 3.21 | | 1.21 – 5.21 | | **0.002** |  |
| Treatment [IMM] * Time.Group [Day 14] | | -0.14 | | -2.48 – 2.20 | | 0.908 |  |
| **Random Effects** | | | | | | |  |
| σ^2^ | | 0.62 | | | | |  |
| τ_00_ _Animal:Experiment_ | | 0.17 | | | | |  |
| τ_00_ _Experiment_ | | 4.03 | | | | |  |
| ICC | | 0.87 | | | | |  |
| N _Animal_ | | 35 | | | | |  |
| N _Experiment_ | | 2 | | | | |  |
| Observations | | 502 | | | | |  |
| Marginal R^2^ / Conditional R^2^ | | 0.057 / 0.879 | | | | |  |
| *contrast* | *Time.Group* | *estimate* | *SE* | *df* | *t.ratio* | *p.value* |  |
| NO - SC | Pre-challenge | 0.840 | 0.273 | 482 | 3.078 | 0.008 |  |
| NO - IMM | Pre-challenge | 0.393 | 0.252 | 482 | 1.559 | 0.163 |  |
| SC - IMM | Pre-challenge | -0.447 | 0.283 | 482 | -1.579 | 0.163 |  |
| NO - SC | Day 9 | 0.408 | 0.327 | 482 | 1.250 | 0.259 |  |
| NO - IMM | Day 9 | 0.950 | 0.302 | 482 | 3.143 | 0.008 |  |
| SC - IMM | Day 9 | 0.542 | 0.336 | 482 | 1.613 | 0.163 |  |
| NO - SC | Day 10 | 0.068 | 0.372 | 482 | 0.182 | 0.856 |  |
| NO - IMM | Day 10 | 0.968 | 0.344 | 482 | 2.812 | 0.015 |  |
| SC - IMM | Day 10 | 0.900 | 0.354 | 482 | 2.544 | 0.024 |  |
| NO - SC | Day 12 | 0.366 | 0.353 | 482 | 1.038 | 0.321 |  |
| NO - IMM | Day 12 | 0.803 | 0.297 | 482 | 2.708 | 0.018 |  |
| SC - IMM | Day 12 | 0.437 | 0.359 | 482 | 1.216 | 0.259 |  |
| NO - SC | Day 14 | 1.697 | 0.423 | 482 | 4.011 | 0.001 |  |
| NO - IMM | Day 14 | 0.980 | 0.309 | 482 | 3.178 | 0.008 |  |
| SC - IMM | Day 14 | -0.716 | 0.420 | 482 | -1.704 | 0.163 |  |

Table 10 Interleukin 17A study 3

|  | | **log IL 17** | | | |  |
| --- | --- | --- | --- | --- | --- | --- |
| *Predictors* | | *Estimates* | | *CI* | *p* |  |
| **Count Model** | | | | | | |
| (Intercept) | | 4.61 | | 3.97 – 5.25 | **<0.001** |  |
| Time.Group [Day 16] | | 0.51 | | -0.43 – 1.46 | 0.288 |  |
| Time.Group [Day 17] | | 1.04 | | 0.03 – 2.05 | **0.044** |  |
| Time.Group [Day 19] | | 1.07 | | -0.04 – 2.18 | 0.058 |  |
| Time.Group [Day 21] | | 1.17 | | 0.16 – 2.18 | **0.023** |  |
| Treatment [IMM] | | 0.16 | | -0.95 – 1.27 | 0.777 |  |
| Time.Group [Day 16] * Treatment [IMM] | | -0.40 | | -1.95 – 1.16 | 0.617 |  |
| Time.Group [Day 17] * Treatment [IMM] | | 0.51 | | -1.12 – 2.14 | 0.541 |  |
| Time.Group [Day 19] * Treatment [IMM] | | -1.73 | | -3.54 – 0.07 | 0.060 |  |
| Time.Group [Day 21] * Treatment [IMM] | | -1.92 | | -3.60 – -0.24 | **0.025** |  |
| **Zero-Inflated Model** | | | | | |  |
| (Intercept) | | 1.01 | | -0.58 – 2.61 | 0.213 |  |
| Treatment [IMM] | | 1.61 | | -0.62 – 3.84 | 0.157 |  |
| Time.Group [Day 16] | | -4.34 | | -6.20 – -2.49 | **<0.001** |  |
| Time.Group [Day 17] | | -3.01 | | -4.44 – -1.59 | **<0.001** |  |
| Time.Group [Day 19] | | -1.78 | | -3.05 – -0.51 | **0.006** |  |
| Time.Group [Day 21] | | -2.68 | | -4.04 – -1.32 | **<0.001** |  |
| **Random Effects** | | | | | |  |
| σ^2^ | | 1.28 | | | |  |
| τ_00_ _Animal_ | | 0.00 | | | |  |
| N _Animal_ | | 10 | | | |  |
| Observations | | 160 | | | |  |
| Marginal R^2^ / Conditional R^2^ | | 0.222 / NA | | | |  |
| *contrast* | *Time.Group* | *estimate* | *SE* | *df* | *t.ratio* | *p.value* |
| NO - IMM | Pre-challenge | -0.160 | 0.565 | 151 | -0.284 | 0.777 |
| NO - IMM | Day 16 | 0.236 | 0.557 | 151 | 0.425 | 0.777 |
| NO - IMM | Day 17 | -0.668 | 0.610 | 151 | -1.095 | 0.459 |
| NO - IMM | Day 19 | 1.574 | 0.730 | 151 | 2.156 | 0.082 |
| NO - IMM | Day 21 | 1.759 | 0.645 | 151 | 2.725 | 0.036 |
