# Supplementary material for: Intramammary Immunisation Provides Short Term Protection Against Mannheimia haemolytica Mastitis in Sheep
Source: Front Vet Sci. 2021 Jun 10;8:659803. doi: 10.3389/fvets.2021.659803 (PMC8222732; doi:10.3389/fvets.2021.659803)
Supplement: Supplementary file 2 [file Table_11.docx]

|  | Pre vaccination conc. (D1) | Post vaccination peak conc. and peak time (D) | Post challenge peak conc. and peak time (D) |
| --- | --- | --- | --- |
| **Interleukin-1β (pg/ml)** |  |  |  |
| Experiment 1 |  |  |  |
| Intramammary vaccination | NA | 1882±1117 **(2)** | NA |
| Subcutaneous vaccination | NA | NA | 6076±3749 **(9)** |
| Controls | NA | NA | 7788±6139 **(9)** |
| Experiment 2 |  |  |  |
| Intramammary vaccination | NA | NA | 2401±5204 **(9)** |
| Controls | NA | NA | 7861±3547 **(9)** |
| Experiment 3 |  |  |  |
| Intramammary vaccination | 486±875 | 3501±2760 **(2)** | 6245±2858 **(17)** |
| Controls | 1715±3927 | 2589±3504 **(2)** | 28058±7088 **(16)** |
| **Interleukin-10 (bu/ml)** |  |  |  |
| Experiment 1 |  |  |  |
| Intramammary vaccination | NA | 3.19 ±1.31 **(4)** | 1.23±1.26 **(9)** |
| Subcutaneous vaccination | NA | NA | 8.67±4.29 **(10)** |
| Controls | NA | NA | 6.27±1.93 **(10)** |
| Experiment 2 |  |  |  |
| Intramammary vaccination | NA | 1.70±2.23 **(2)** | 0.93±0.65 **(9)** |
| Controls | NA | NA | 8.79±3.98 **(9)** |
| Experiment 3 |  |  |  |
| Intramammary vaccination | NA | NA | 11.09±4.**38 (17)** |
| Controls | NA | NA | 4.44±3.09 **(17)** |
| **Interleukin-17A (pg/ml)** |  |  |  |
| Experiment 1 |  |  |  |
| Intramammary vaccination | NA | 1317±2067 **(4)** | 1359±1086 **(9)** |
| Subcutaneous vaccination | NA | 507±790 **(2)** | 1913±2326 **(9)** |
| Controls | 629±679 | NA | 1725±2240 **(14)** |
| Experiment 2 |  |  |  |
| Intramammary vaccination | NA | NA | NA |
| Controls | NA | NA | 540±989 **(9)** |
| Experiment 3 |  |  |  |
| Intramammary vaccination | 364±1066 | NA | 580±1094 **(17)** |
| Controls | NA | NA | 305±231 **(21)** |

**Supplementary table 11.** Concentration of milk cytokines above the baseline pre vaccination, post vaccination peak and post challenge peak. For each experimental group average concentration and standard deviation are shown.
