# Supplementary figures and images for: Intramammary Immunisation Provides Short Term Protection Against Mannheimia haemolytica Mastitis in Sheep
Source: Front Vet Sci. 2021 Jun 10;8:659803. doi: 10.3389/fvets.2021.659803 (PMC8222732; doi:10.3389/fvets.2021.659803)

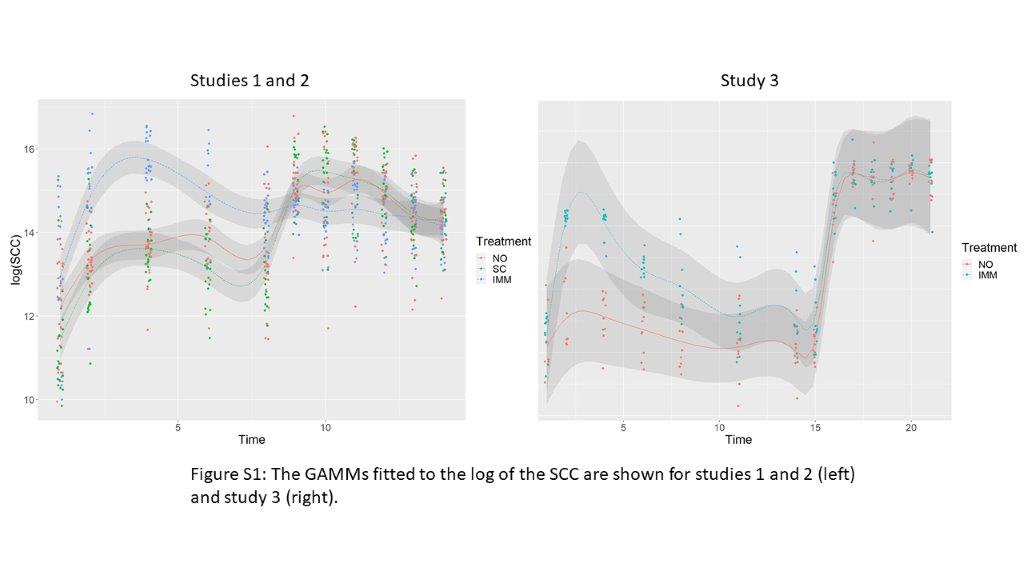

Supplement: Supplementary file 3 [file Image_1.JPEG]
